# Simultaneous Improvement of Bendability and Passive Daytime Radiative Cooling Performance in Multilayer Alumina Fiber Membranes

**DOI:** 10.3390/ma19132914

**Published:** 2026-07-07

**Authors:** Yating Zhuang, Chongyang Fu, Benxing Guo, Weihao Zhai, Xueting Ren, Depeng Fu, Xianchao Li, Guangzheng Wang, Qizheng Li, Yidan Xiao, Shuye Zhang, Hanbin Wang, Xiaoxiong Wang

**Affiliations:** 1 College of Physics Science, Qingdao University, Qingdao 266071, China; 2 Qingdao Xinanqu Refractories Co., Ltd., Qingdao 266000, China; 3 State Key Laboratory of Advanced Welding and Joining, Harbin Institute of Technology, Harbin 150001, China; 4 Microsystem & Terahertz Research Center, China Academy of Engineering Physics, Chengdu 610200, China

**Keywords:** passive daytime radiative cooling, alumina nanofiber membrane, layered interface, multiple-neutral-axis mechanism, solar reflectance, thermal barrier effect

## Abstract

Passive daytime radiative cooling (PDRC) materials require high solar reflectance and high atmospheric window emissivity. However, high solar reflectance achieved by scattering strategies often relies on porous structures, which can compromise the material’s mechanical reliability. To address this trade-off, we develop a layered alumina nanofiber membrane (LANM) by dual-nozzle electrospinning with programmed alternating deposition, in which alternating deposition and subsequent removal of alumina precursor layers and sacrificial polyvinyl alcohol (PVA) interlayers generate a continuously layered architecture with periodic interfaces and interlayer air gaps. This interfacial geometric design enables simultaneous regulation of solar-band scattering and bending load transfer within a single alumina system. Because photon flux attenuates with depth, shallow interfaces contribute more strongly than deeper ones; therefore, the micro-layered architecture enhances scattering while maintaining high emissivity in the atmospheric window. In outdoor testing, LANM achieved a maximum sub-ambient temperature reduction of ~5.8 °C, representing a further improvement of about 2.4 °C compared to Monolithic alumina nanofiber (ANM). Moreover, interlayer interfaces induce a multiple-neutral-axis mechanism and segmented stress transfer, thereby improving bending deformability rather than load-bearing strength.

## 1. Introduction

As the global climate crisis intensifies, the rapidly increasing demand for space cooling has become a critical energy and environmental challenge. Space cooling, including air conditioners and electric fans, accounts for nearly 20% of the total electricity used in buildings worldwide, and this demand is expected to continue increasing under global warming [1]. Passive daytime radiative cooling (PDRC) provides a zero-energy cooling pathway by simultaneously reflecting solar irradiation in the 0.3–2.5 μm range and emitting thermal radiation through the atmospheric window of 8–13 μm [2,3,4]. When the net radiative cooling power exceeds the absorbed solar energy and non-radiative heat gain, a surface can spontaneously reach a temperature below ambient air even under direct sunlight. Therefore, PDRC has attracted increasing attention for building thermal management, mitigation of urban heat island effects, and reduction of cooling-related carbon emissions [5,6,7].

Existing PDRC materials can generally be divided into polymers, cellulose-based materials, organic/inorganic composites, and inorganic materials [8]. Among them, inorganic systems are particularly attractive for long-term outdoor applications because of their excellent weather resistance, UV-aging resistance, chemical stability, and thermal stability [9]. Representative inorganic or inorganic-containing PDRC materials include HfO_2_/SiO_2_ multilayer photonic structures, ultra-white BaSO_4_ coatings, and hierarchically porous Al_2_O_3_/glass ceramics, which have demonstrated the feasibility of achieving high solar reflectance and efficient daytime cooling through optical structural design [10,11,12]. Recently, Wang et al. reported a high-performance double-layer radiative cooling coating based on PVDF/PDMS layers filled with Al_2_O_3_ and SiO_2_ particles, achieving a solar reflectance of 98%, an atmospheric-window emissivity of 0.95, and a maximum sub-ambient temperature reduction of 7.1 °C under direct midday sunlight [13]. Nevertheless, achieving a balance between high-efficiency photothermal regulation and mechanical deformability remains challenging [14,15]. For dielectric PDRC materials, high solar reflectance mainly originates from multiple scattering caused by the refractive-index contrast between the solid framework and the air [5,16,17]. Increasing porosity and optimizing pore-size distribution can enhance Mie scattering and solar-band backscattering [18,19,20,21], but excessive pores may also act as mechanical defect sources, leading to stress concentration, crack initiation, and reduced structural reliability under bending or handling [22,23,24,25]. This optical–mechanical trade-off is particularly evident in porous inorganic PDRC materials, which often need high porosity for strong scattering but sufficient integrity for practical use [26,27,28].

Electrospinning offers an effective route to fabricate ceramic nanofiber membranes with interconnected fibrous networks, nanoscale fiber diameters, and macroscopic flexibility [29,30,31,32]. Recent studies have shown that electrospun inorganic nanofiber membranes can combine thermal stability, fire resistance, and functional performance in flexible film forms [33,34,35]. However, conventional monolithic electrospun inorganic membranes generally lack controllable internal interfaces that can simultaneously increase optical scattering pathways and regulate bending stress transfer [10,36]. Based on this consideration, we propose a layered alumina nanofiber membrane (LANM) prepared by dual-nozzle electrospinning with programmed alternating deposition of alumina precursor layers and sacrificial PVA layers. After calcination, the removal of PVA generates periodic interlayer interfaces and air gaps within a single alumina fibrous system. Optically, the layered architecture increases effective scattering interfaces while maintaining high atmospheric-window emissivity. Mechanically, the weak interlayer interfaces promote segmented stress transfer and a multiple-neutral-axis bending mode, which improves bendability and delays catastrophic fracture [37,38,39,40]. This work therefore provides an interfacial-structure design strategy for simultaneously regulating radiative-cooling behavior and bending deformability in inorganic fibrous PDRC membranes.

## 2. Experimental

### 2.1. Materials

Polyvinylpyrrolidone (PVP, Mw = 1,300,000) and polyvinyl alcohol (PVA, Mw = 89,000, 99% hydrolyzed) were purchased from Shanghai Aladdin Biochemical Technology Co., Ltd. (Shanghai, China) Aluminum chloride hexahydrate and acetic acid were obtained from China National Pharmaceutical Group Chemical Reagent Co., Ltd. (Beijing, China) Anhydrous ethanol was purchased from Tianjin Fuyu Fine Chemical Co., Ltd. (Tianjin, China). Aluminum isopropoxide and tartaric acid were purchased from Shanghai McLean Biochemical Technology Co., Ltd. (Shanghai, China). Deionized water (18.2 MΩ·cm) was used throughout. All chemicals were used as received.

### 2.2. Preparation of Layered Alumina Nanofiber Membranes

Monolithic ANM and LANM were fabricated by dual-nozzle co-electrospinning combined with programmed alternating deposition. The alumina precursor solution was prepared from aluminum chloride hexahydrate, deionized water, ethanol, aluminum isopropoxide, tartaric acid, and glacial acetic acid in a mass ratio of 1:3:4.1:2.1:0.11:1.48, followed by addition of 5 wt% PVP. The sacrificial solution was a 10 wt% PVA aqueous solution prepared at 80 °C.

The alumina precursor and PVA solutions were delivered through 5 mL syringes (needle inner diameter ~0.13 mm) at 1.0 and 0.8 mL·h^−1^, respectively, under 18 kV. Fibers were collected 20 cm from the needle tip on a drum rotating at 300 r/min. Electrospinning was carried out at 33 °C and 30% relative humidity. The as-spun membranes were calcined in air at 1200 °C for 2 h to remove organic components and obtain Monolithic ANM and LANM.

To ensure a comparable alumina loading among different samples, the total feeding volume of the alumina precursor solution was kept the same for Monolithic ANM and LANM. The layer number was regulated by dividing the alumina precursor deposition into different programmed segments, while PVA was introduced between adjacent alumina precursor segments as a sacrificial spacer.

For the Monolithic ANM, 5 mL of alumina precursor solution was continuously electrospun without PVA deposition. For the *N*-layer LANM, the same total volume of alumina precursor solution, 5 mL, was divided into N alumina deposition segments, and PVA sacrificial layers were deposited between neighboring alumina precursor segments. Taking the five-layer LANM as an example, the deposition sequence was Al_2_O_3_ precursor/PVA/Al_2_O_3_ precursor/PVA/Al_2_O_3_ precursor/PVA/Al_2_O_3_ precursor/PVA/Al_2_O_3_ precursor. The total PVA feeding volume was 5 mL.

The actual solid deposition amount of each individual layer was not separately measured because the electrospun jet undergoes solvent evaporation during flight and the sacrificial PVA layers are removed during calcination. The heating rate for calcination was 1 °C min^−1^ and 5 °C min^−1^, followed by holding at 1200 °C for 2 h and naturally cooling to room temperature.

### 2.3. Structural and Compositional Characterization

Morphology was characterized by FE-SEM (JSM-7800F, JEOL, Tokyo, Japan), and fiber diameters were analyzed using ImageJ (Version 1.54p, National Institutes of Health, Bethesda, MD, USA). Elemental distributions were measured by SEM-equipped EDS. Phase composition was analyzed by XRD (SmartLab 3 kW, Rigaku, Tokyo, Japan). Chemical bonds were characterized by FT-IR (Nicolet iS50, Thermo Fisher Scientific, Waltham, MA, USA). Thermal decomposition of precursor films was examined by TGA (TGA 2, Mettler Toledo, Greifensee, Switzerland).

### 2.4. Mechanical Properties and Finite Element Simulation

Bending properties were measured using an electromagnetic compensation three-point bending system (TPS 4585A, assembled in-house, Qingdao University, Qingdao, China), and full bending stress–strain curves were recorded under identical loading and geometric conditions. The rectangular membrane specimens had a length of 15 mm, a width of 6–8 mm, and a thickness of 0.18–0.23 μm. The support span was 5 mm. At least 3 independent specimens were tested for each sample. The nominal bending stress and nominal bending strain were calculated using the standard three-point bending equations. Because LANM contains interlayer gaps and may allow partial interlayer sliding, the calculated stress and strain are regarded as nominal values for comparing the apparent bending response under identical testing conditions, rather than true local stress and strain within individual sublayers. Representative original load–displacement curves are provided in Figure S6. Finite element analysis was performed in Abaqus 2021 to qualitatively compare the through-thickness stress distribution of the monolithic and layered models under bending. All alumina fibrous layers were treated as linear elastic materials with identical material parameters. The monolithic model was constructed as a continuous beam, whereas the layered model was divided into five sublayers with the same total thickness. A three-point bending condition was applied using two bottom supports and a vertical displacement at the mid-span position. The interlayer interfaces were modeled as surface-to-surface contact boundaries that allowed tangential sliding while preventing normal penetration. The models were meshed with refined solid elements through the thickness direction, and the longitudinal normal stress component S11 was extracted for comparison.

For cross-material validation, multilayer assemblies were also constructed using mica plates, SiO_2_ nanofiber membranes, and PZT nanofiber membranes, with A4 paper as a supplementary control. SiO_2_ and PZT monolayers were prepared by conventional electrospinning and calcination, then physically stacked, whereas mica plates and A4 paper were directly laminated layer by layer.

### 2.5. Optical Properties

Solar reflectance (0.3–2.5 μm) was measured using a UV/Visible/near-infrared (NIR) spectrophotometer (PE Lambda 750, Waltham, MA, USA). Mid-infrared emissivity (8–13 μm) was measured using an FT-IR spectrometer (Nicolet iS50, Thermo Fisher Scientific, Waltham, MA, USA) equipped with an integrating sphere.

### 2.6. Radiative Cooling Test

Outdoor cooling tests were carried out in Qingdao, China, under clear-sky conditions from 10:30 to 17:30 on 6 December 2025. Each sample had an exposed area of 8 cm × 8 cm. A polystyrene foam box covered with aluminum foil was used as the test box to reduce heat exchange with the surroundings and reflect environmental radiation. An infrared-transparent polyethylene film was placed above the samples to reduce convective heat exchange while allowing mid-infrared radiation to pass through. The thermocouple probe was positioned at the center of the test box to monitor the sample temperature, while the ambient temperature was recorded simultaneously near the testing setup. Sample and ambient temperatures were monitored using thermocouples (UT-T12, UNI-T, Dongguan, China) and recorded by a nanovoltmeter (Keithley 2182, Keithley Instruments, Cleveland, OH, USA). Solar irradiance was measured with a solar radiometer (TES 1333R, TES Electrical Electronic Corp., Taipei, Taiwan), while ambient humidity and wind speed were recorded simultaneously. In addition to the maximum temperature difference, the average temperature difference during the testing period was also calculated. A repeated outdoor test under clear-sky conditions was further provided in the Supporting Information.

## 3. Results and Discussion

### 3.1. Construction and Characterization of Multilayer Structures

**Figure 1:**
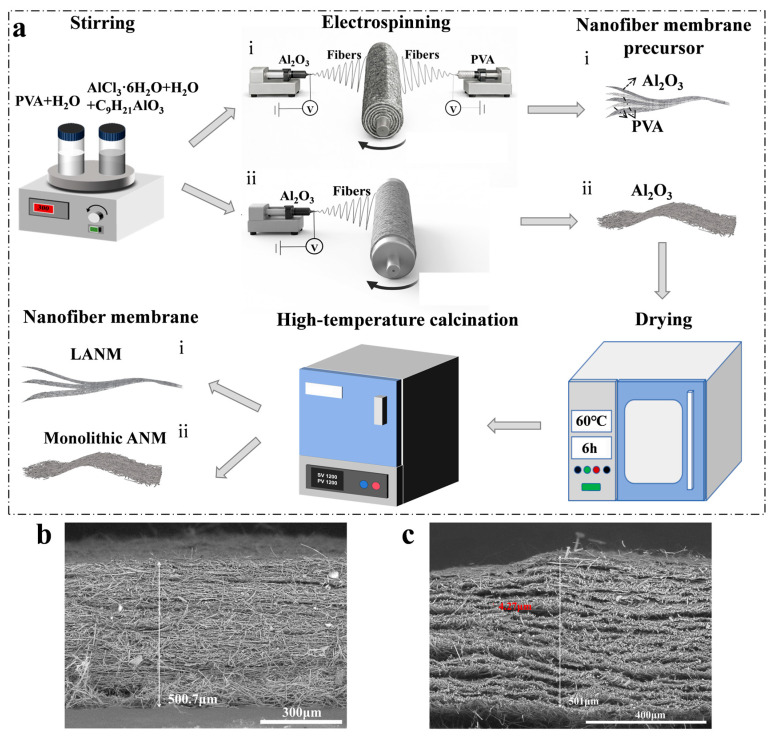
Fabrication and structural characterization of Monolithic ANM and LANM. (**a**) Schematic illustration of the preparation process for Monolithic ANM and LANM, where (**i**) represents the multilayer process flow and (**ii**) represents the single-layer process flow (**b**) Cross-sectional SEM image of Monolithic ANM. (**c**) Cross-sectional SEM image of LANM.

To construct controllable interlaminar interfaces within an inorganic nanofiber network, dual-nozzle co-electrospinning with programmed sequential deposition was employed (Figure 1a). This deposition strategy allows the interfacial structure to be introduced during membrane formation, rather than by post-assembly lamination. Therefore, the final membrane maintains an integrated fibrous network while containing designed internal interfaces. The Al_2_O_3_ precursor jet and sacrificial PVA jet were alternately deposited onto the collection region to form an alternately stacked precursor architecture. The PVA layers act as temporary spacers between adjacent alumina precursor layers. After calcination, their removal creates interlayer gaps without introducing an additional solid component into the final ceramic membrane. After drying and high-temperature calcination, the PVA component was removed, yielding the layered alumina nanofiber membrane (LANM). In contrast, single-nozzle continuous deposition of the Al_2_O_3_ precursor produced a uniform fibrous membrane without discernible internal interfaces; after calcination, this sample became a monolithic alumina nanofiber membrane (Monolithic ANM).

The cross-sectional SEM images in Figure 1b,c clearly reveal the structural difference between the two configurations. The Monolithic ANM exhibits a continuous and compact fibrous cross-section, whereas the LANM shows a distinct layered morphology with periodically distributed interlaminar gaps. Although the total thicknesses remain comparable, the LANM is subdivided into thinner units, with an average sublayer thickness of approximately 4.27 μm, indicating that the layered architecture arises from deposition-mode regulation rather than a simple increase in film thickness. This comparison is important because it allows the optical and mechanical differences between Monolithic ANM and LANM to be mainly attributed to the internal layered architecture instead of the overall membrane thickness. The periodic gaps also provide additional internal boundaries for light scattering and stress redistribution.

**Figure 2:**
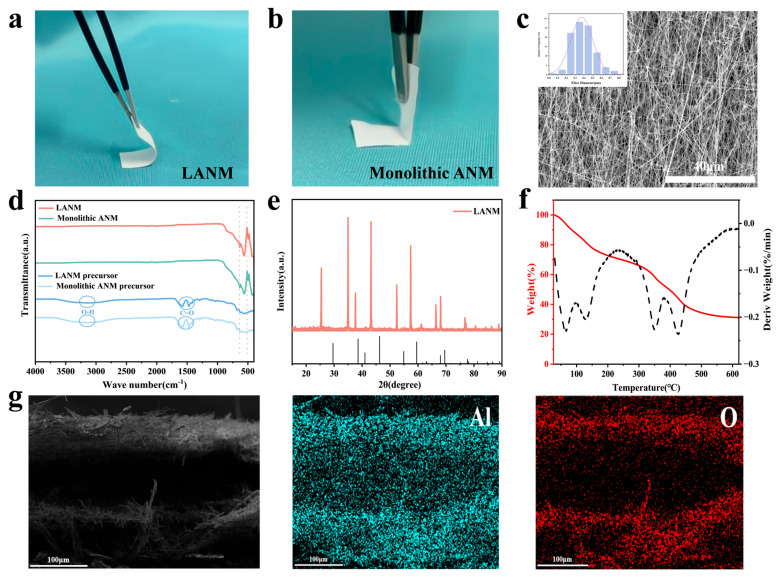
Morphological, structural, and compositional characterization of Monolithic ANM and LANM. (**a**) Macroscopic flexibility demonstration of LANM. (**b**) Macroscopic flexibility demonstration of Monolithic ANM. (**c**) SEM image of LANM and corresponding fiber diameter distribution. (**d**) FT–IR spectra of precursor and calcined samples for Monolithic ANM and LANM. (**e**) XRD pattern of LANM after calcination. (**f**) Thermogravimetric (TG) and derivative thermogravimetric (DTG) curves of LANM precursor. (**g**) Cross-sectional SEM image and corresponding EDS elemental mapping (Al and O) of LANM.

The calcined LANM exhibits a uniform appearance and maintains good structural integrity with pronounced macroscopic flexibility (Figure 2a), whereas the Monolithic ANM shows limited deformation under the same operation (Figure 2b). The improved bendability of LANM is mainly related to the subdivision of the brittle alumina network into thinner fibrous sublayers. During bending, these sublayers can undergo slight relative displacement, which reduces continuous through-thickness strain accumulation. At the microscopic scale, LANM retains a continuous and porous fibrous network (Figure 2c). Such an interconnected fibrous structure is beneficial for maintaining membrane integrity after calcination, because external deformation can be accommodated by local fiber bending and rearrangement instead of immediate through-thickness cracking. Fiber diameter statistics indicate that the precursor fibers possess an average diameter of 1.74 μm (Figure S1). After calcination, the average diameter decreases to 0.37 μm due to fiber densification (Figure 2c); Monolithic ANM follows a comparable trend (Figures S2 and S3). The similar fiber-diameter evolution indicates that the difference between Monolithic ANM and LANM mainly originates from the macroscopic layer arrangement rather than from a change in the intrinsic fiber morphology. The reduced fiber diameter and interconnected network provide abundant scattering pathways within the porous structure. Meanwhile, the submicron fiber size is comparable to the characteristic wavelength range of solar irradiation, which is favorable for enhancing multiple scattering in the solar spectrum.

The FT–IR spectra (Figure 2d) show that the characteristic absorption peaks of the precursor at ~3400 cm^−1^ (O–H) and ~1660 cm^−1^ (C=O) are significantly weakened after calcination, while a broad band below 1000 cm^−1^ associated with Al–O vibrations becomes dominant. This confirms the effective removal of organic components and the formation of an inorganic alumina-based framework after high-temperature treatment. The XRD pattern (Figure 2e) confirms the formation of α-Al_2_O_3_ after calcination (JCPDS No. 10-0173). The formation of thermally stable α-Al_2_O_3_ provides the structural basis for the subsequent flame-exposure stability of LANM. The TG–DTG curves (Figure 2f) show that the main weight loss occurs between 280 and 420 °C, followed by stabilization at higher temperatures. EDS mapping (Figure 2g) shows a uniform distribution of Al and O elements across the cross-section of LANM.

### 3.2. Synergistic Optical Enhancement and Radiative Cooling

To clarify the role of sublayer scale in scattering-interface activation, the solar reflectance of Monolithic ANM and LANM was compared at a comparable total thickness. This design minimizes the influence of thickness variation on reflectance and allows a more direct comparison of the effect of interfacial architecture. The reflectance difference is therefore mainly associated with the sublayer-scale and interface-density modulation introduced by the layered architecture, rather than with the overall film thickness.

**Figure 3:**
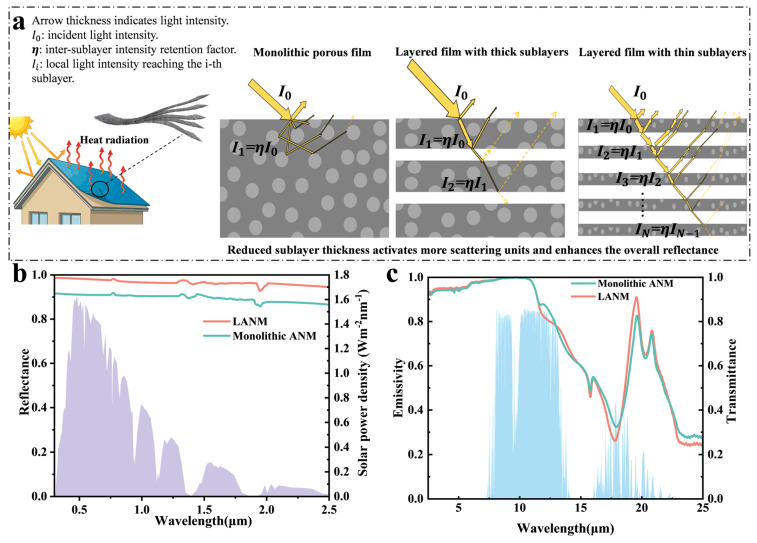
Optical properties and spectral response of Monolithic ANM and LANM. (**a**) Schematic illustration of light attenuation and reflected output in a monolithic porous film, a layered film with thick sublayers, and a layered film with thin sublayrs. (**b**) UV–Vis–NIR reflectance spectra of Monolithic ANM and LANM in the 0.3–2.5 μm range, with the AM 1.5G solar irradiance shown as the purple shaded background. (**c**) Mid-infrared emissivity spectra of Monolithic ANM and LANM in the atmospheric-window region, with atmospheric-window transmittance shown as the blue shaded background.

As shown in Figure 3a, incident light in Monolithic ANM undergoes multiple random scatterings within the continuous porous framework, and the local light intensity decays with penetration depth, which can be phenomenologically expressed as

(1) $$I\left(z\right)=I_{0}exp\left(-\frac{z}{l_{eff}}\right)$$

where $I_{0}$ is the incident optical intensity and $l_{eff}$ is the effective attenuation length associated with multiple scattering and internal losses. Under this continuous attenuation mode, the upper region consumes a substantial fraction of the incident photon flux, leaving deeper scattering units in a low-intensity regime and limiting their contribution to macroscopic backscattering. As a result, simply increasing the thickness of a continuous monolithic membrane does not necessarily lead to proportional improvement in solar reflectance, because the deeper region may become optically less active.

By contrast, LANM divides the continuous attenuation path into multiple sublayers and redistributes the through-thickness optical intensity. In Figure 3a, arrow thickness indicates light intensity, η denotes the inter-sublayer intensity retention factor, and I_i_ denotes the local light intensity reaching the i-th sublayer. As the sublayer thickness decreases, the intralayer propagation distance is shortened, enabling more optical intensity to be retained in deeper regions. Thus, more internal regions can participate in effective backscattering before the incident light is fully attenuated. At the same time, the number of air/Al_2_O_3_ interfaces increases across the thickness direction, providing more effective scattering sites. Consequently, reduced sublayer thickness simultaneously suppresses deep-region intensity depletion and activates more through-thickness scattering units, thereby strengthening cumulative backscattering and enhancing the solar reflectance of LANM. The enhanced solar reflectance of LANM is attributed to the combined effect of the porous alumina fibrous network and the additional internal interfaces introduced by the layered structure. It should be noted that this interpretation is qualitative, because the present work does not directly fit the scattering coefficient or separately quantify the contribution of each structural parameter. Therefore, the interlayer interfaces are not claimed to be the only factor responsible for the reflectance enhancement. Other structural factors, such as porosity, apparent density, mass per unit area, fiber packing, and surface roughness, may also influence the optical response [27]. Nevertheless, Monolithic ANM and LANM were prepared from the same alumina precursor system and exhibited similar fiber morphology and comparable total thickness, suggesting that the increased number of internal air/Al_2_O_3_ interfaces is an important structural factor contributing to the higher solar reflectance of LANM.

Experimental results show that, compared with Monolithic ANM, LANM exhibits a higher reflectance in the 0.3–2.5 μm range, with the average solar reflectance increasing from 87% to 95% (Figure 3b). In contrast, LANM maintains a stable high emissivity in the atmospheric window region (Figure 3c), with an average emissivity of ~92% and little variation between samples. This indicates that the mid-infrared emission is mainly governed by the intrinsic vibrational absorption of alumina and the porous fibrous framework, rather than being strongly disturbed by the layered arrangement. These results indicate that the layered geometry mainly optimizes scattering in the solar band, while the mid-infrared emission remains dominated by Al_2_O_3_ lattice vibrations, enabling LANM to simultaneously satisfy the spectral requirements of high solar reflectance and high atmospheric-window emissivity for PDRC. Therefore, the layered design improves the solar-reflection component of PDRC without sacrificing the thermal-emission requirement in the atmospheric window.

Although the absolute solar reflectance and maximum cooling temperature reduction of LANM are lower than those of the reported double-layer PVDF/PDMS-based PDRC coating [14], the present work focuses on a different design target. LANM is a single-component inorganic alumina fibrous membrane, in which internal multilayer interfaces simultaneously enhance solar scattering, improve bendability, and provide flame-exposure stability. Therefore, its advantage lies in the integration of radiative cooling capability with inorganic structural robustness rather than in achieving the highest reported cooling temperature reduction.

**Figure 4:**
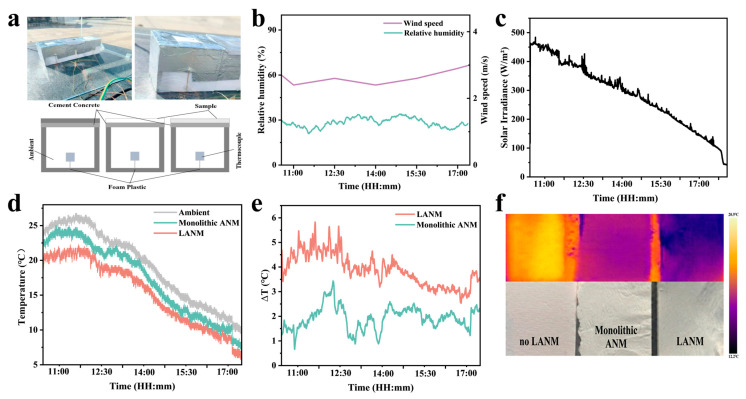
Outdoor radiative cooling performance testing. (**a**) Physical and schematic diagrams of the test setup. (**b**) Wind speed and relative humidity on the test day. (**c**) Solar irradiance on the test day. (**d**) Schematic layout of on-site temperature monitoring. (**e**) Temperature difference curves for samples with different numbers of layers. (**f**) Infrared thermal imaging comparison of no LANM, Monolithic ANM, and LANM.

To evaluate actual cooling performance, an outdoor test setup (Figure 4a) was constructed that continuously monitored key parameters, including solar irradiance, ambient temperature, humidity, and wind speed (Figure 4b,c). The temperature difference between the sample surface and ambient temperature, ΔT = T_ambient_ − T_sample_, is shown in Figure 4d,e. Under direct diurnal solar irradiation, all samples exhibited sustained positive ΔT values, confirming their net daytime cooling capability. Cooling effectiveness increased with the number of layers. In the main outdoor test on 6 December 2025, LANM film (*N* = 5) achieved a maximum ΔT of 5.8 °C, representing an improvement of approximately 2.4 °C over Monolithic ANM. To avoid relying only on the maximum temperature difference, the average ΔT over the measurement period was also calculated. The average ΔT values of LANM and Monolithic ANM were 3.79 and 1.92 °C, respectively. A repeated outdoor test under clear-sky conditions was further conducted on 7 December 2025, and the results are provided in Figure S5. In the repeated test, LANM and Monolithic ANM showed maximum ΔT values of 5.0 and 2.9 °C, respectively, with corresponding average ΔT values of 3.55 and 1.85 °C. These repeated measurements confirm that LANM consistently exhibits a larger sub-ambient cooling effect than Monolithic ANM under the same outdoor testing conditions.

Further infrared thermal imaging comparisons visually demonstrate the cooling effect (Figure 4f). Upon reaching steady-state thermal equilibrium under direct solar irradiation, the samples placed on the test substrate were evaluated. The temperature in the LANM-covered area was consistently lower than that in the blank control area, with the lowest temperature observed in the region corresponding to LANM. Field testing under direct sunlight confirms that LANM achieves stable net daytime cooling. Although the maximum temperature reduction is used to compare different samples, the overall temporal trend also supports the improved cooling capability of the layered membrane. Its multilayer structure enhances solar reflectivity while maintaining high atmospheric window emissivity, enabling greater temperature reduction and superior sub-ambient cooling performance.

To further contextualize these results, the performance of LANM was compared with representative inorganic or ceramic-fiber-based PDRC systems, as summarized in Table S1 (Supporting Information). LANM exhibits a competitive maximum sub-ambient cooling temperature reduction of 5.8 °C. Although some reported systems show higher absolute cooling temperature reductions or cooling powers, the present LANM provides a distinct, balanced combination of radiative cooling capability, a single-component inorganic alumina composition, and improved bending deformability. Therefore, rather than claiming record-high cooling metrics, this work highlights the structural multifunctionality and balanced reliability enabled by the multilayer alumina fibrous architecture.

### 3.3. Mechanism of Improved Bendability

**Figure 5:**
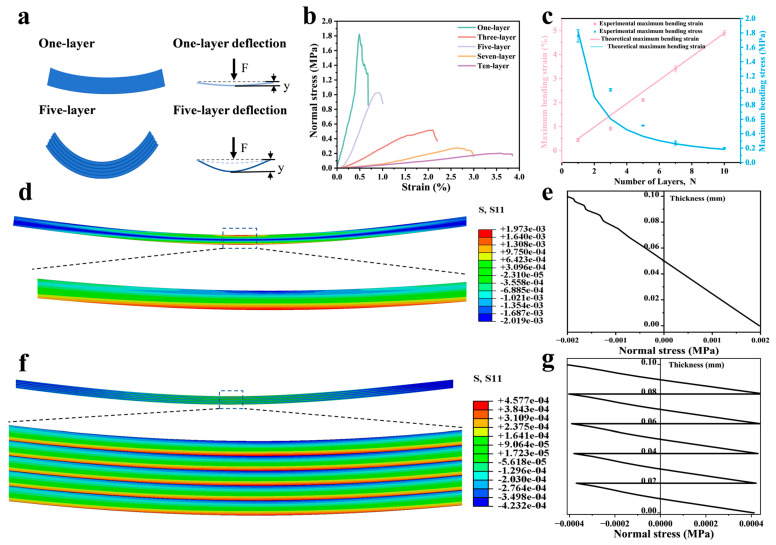
Bending behavior assessment of Monolithic ANM and LANM: (**a**) Schematic comparison of bending deformation modes for the one-layer sample (Monolithic ANM) and layered samples. (**b**) Nominal bending stress–strain curves for the one-layer sample (*N* = 1), three-layer LANM (*N* = 3), and five-layer LANM (*N* = 5). (**c**) Dependence of the experimental and theoretical maximum bending strain and maximum bending stress on layer number N, with dashed lines showing the theoretical scaling and extrapolated trends toward large *N*. FEA contour plots of the longitudinal normal stress component S11 for (**d**) the one-layer model and (**f**) the five-layer model. Through-thickness S11 profiles for (**e**) the one-layer model and (**g**) the five-layer model.

Three-point bending tests were performed on alumina nanofiber membranes with different layer numbers (Figure S4). As illustrated in Figure 5a, the one-layer sample (Monolithic ANM, *N* = 1) bends around a single neutral plane, whereas layered samples develop local neutral planes within individual sublayers. This structural difference reduces the effective bending thickness of each load-bearing unit and weakens the continuity of tensile strain across the full membrane thickness. This geometry redistributes bending strain across the thickness, suppresses stress continuity, and permits larger curvature before failure. Therefore, the layered structure is discussed here in terms of improved bendability and bending tolerance, rather than general mechanical strengthening. The corresponding bending stress–strain curves are shown in Figure 5b. The one-layer sample exhibits typical brittle fracture, with a maximum bending strain of ~0.5% and a maximum bending stress of ~1.8 MPa. As the layer number increases, the maximum bending strain rises markedly, and the failure mode gradually changes from abrupt instability to a more progressive process. For the five-layer LANM (*N* = 5), the maximum bending strain reaches ~2.1%, while the maximum nominal bending stress decreases to ~0.5 MPa. The decrease in nominal bending stress indicates that LANM should not be interpreted as mechanically stronger in terms of load-bearing capacity. Instead, the main mechanical advantage is its ability to tolerate larger bending deformation before catastrophic failure. Minor fluctuations and a short plateau appear near the peak, indicating staged stiffness change and load redistribution during failure. This trend is further supported by the macroscopic bending morphology and the dynamic fracture process (Video S1). Monolithic ANM fractures prematurely, whereas LANM sustains deformation for a longer duration, which is consistent with the delayed fracture enabled by interlayer interfaces.

To highlight the layer-number dependence, the experimentally extracted maximum bending strain and maximum bending stress are replotted against *N* in Figure 5c, together with the theoretical scaling and extrapolated trends. As *N* increases, the apparent maximum bending strain increases monotonically, whereas the maximum bending stress decreases continuously. This opposite evolution indicates that layer subdivision improves structural bendability while lowering the nominal stress level sustained by the membrane.

The mechanism can be understood as through-thickness stress transfer during bending. In the initial small-deflection elastic regime, the one-layer sample can be approximated as an Euler–Bernoulli beam, and the maximum outer-surface bending stress is

(2) $$\sigma_{mono}=E\cdot \varepsilon =\frac{E\cdot H}{2\rho }$$

where *E* is the equivalent elastic modulus, *H* is the total thickness, and *ρ* is the bending radius of curvature. In the layered structure, weak interfaces created by interlayer air gaps disrupt deformation continuity and cause each sublayer to bend around its own local neutral plane, thereby activating the multiple-neutral-axis mechanism. Under ideal decoupling and free sliding, when the total thickness *H* is divided into *N* identical sublayers, the maximum local bending stress in each sublayer becomes

(3) $$\sigma_{lami}=\frac{1}{N}\sigma_{mono}$$

and the apparent maximum bending strain at failure follows

(4) $$\varepsilon_{max}\left(N\right)=N\varepsilon_{f}$$

where $\varepsilon_{f}$ is the intrinsic fracture strain of a single sublayer. The derivation of Equation (4) is provided in the Supporting Information. Equations (3) and (4) predict that increasing *N* should simultaneously reduce the maximum local bending stress and increase the apparent structural bending limit. The theoretical lines in Figure 5c represent these ideal trends and their extrapolation toward larger *N*.

The experiments follow the same overall trend, but do not reach the fully decoupled limit. For example, the peak bending stress of the five-layer LANM is ~0.5 MPa, corresponding to ~28% of that of the one-layer sample (~1.8 MPa), rather than the ideal value of 20%. Likewise, the measured maximum bending strain increases with N but remains below the ideal linear extrapolation. These deviations indicate that adjacent fibrous sublayers are not fully independent. Instead, partial mechanical coupling is retained through interfacial friction and nanoscale fiber interlocking. This partial coupling is beneficial: it relaxes local strain concentration through the multiple-neutral-axis mechanism, while preserving sufficient interfacial shear transfer to maintain structural integrity.

Finite element simulations further support this interpretation (Figure 5d–g). The longitudinal normal stress component S11, rather than the von Mises stress, was extracted to evaluate the bending-induced tensile and compressive stress distribution. The one-layer model exhibits a continuous linear gradient of S11 through the thickness, consistent with monolithic beam bending. By contrast, the five-layer model shows clear stress reset at interlayer interfaces, producing a segmented sawtooth S11 profile. Each sublayer retains an approximately linear internal gradient, but the stress level changes abruptly across adjacent interfaces. This sawtooth through-thickness S11 distribution supports the proposed multiple-neutral-axis mechanism and is consistent with the experimentally observed increase in bending deformability and decrease in nominal peak stress.

To test the generality of this layer-subdivision mechanism, multilayer assemblies were further constructed using mica plates, SiO_2_ nanofiber membranes, and PZT nanofiber membranes, with A4 paper as a supplementary control. Their bending behavior was evaluated in Figures S7–S11. All systems show the same trend with increasing layer number: lower maximum bending stress and higher maximum bending strain. These results indicate that the multiple-neutral-axis mechanism enables segmented stress transfer across the thickness and provides a broadly applicable strategy for improving bendability in flexible inorganic thin films [38].

### 3.4. Fire Resistance

**Figure 6:**
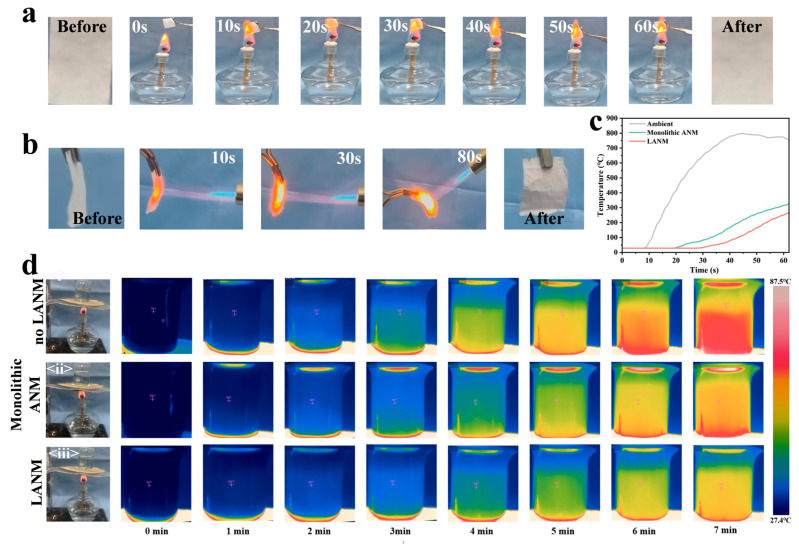
Fire resistance and thermal insulation performance. (**a**) The LANM before and after 60 s exposure to the alcohol lamp. (**b**) The LANM before and after 80 s exposure to a flame torch. (**c**) Back temperature–time curves for Monolithic ANM and LANM. (**d**) Infrared thermal imaging of no LANM, Monolithic ANM, and LANM after 7 min immersion in a hot water bath.

As a fully inorganic film designed for passive radiative cooling applications, LANM must withstand extreme conditions, including high-temperature environments and occasional thermal shocks during practical use. While material temperatures on building exteriors exposed to direct sunlight typically do not reach flame temperatures, PDRC materials must still exhibit fundamental fire resistance and thermal barrier capabilities under conditions such as localized heat-source contact, fire propagation, or high-heat-flux-density irradiation. This prevents structural failure or accelerated aging. Further investigation examined the response of LANM to direct flame exposure and sustained heating, comparing it with common polymer films (PA6, TPU) and high-purity aluminum foil substrates (99.99%) (Figure S12 and Figure 6).

Under direct exposure to an alcohol lamp flame, PA6 and TPU exhibited significant softening and melting within a short timeframe, followed by perforation and membrane collapse (Figure S12a,b), demonstrating the rapid failure behavior typical of polymer films under flame impact. This contrast mainly originates from the difference in chemical composition, since organic polymer chains are vulnerable to softening, melting, and decomposition at elevated temperatures. In contrast, no open-flame combustion or perforation failure was observed in LANM during the same exposure time, and the overall morphology of the sample remained stable (Figure 6a). The stable morphology is attributed to the nonflammable alumina framework formed after high-temperature calcination. Under stronger hot airflow conditions, we conducted exposure tests using a flame torch. LANM was placed between the flame and high-purity aluminum foil (Figure S12c). Post-exposure observations focused on film integrity and aluminum foil morphology. Unlike the directly heated areas, the LANM-covered foil region showed no melting or perforation. This result indicates that LANM can partially block direct heat transfer. Even after prolonged exposure to a torch, LANM maintained structural integrity (Figure 6b). These results indicate that LANM exhibits good morphology retention under direct flame exposure and high-heat-flux impact.

The layered structure also contributes to a thermal-barrier effect under the present testing conditions. The interlayer air gaps and discontinuous solid pathways may increase the apparent thermal resistance across the membrane thickness and delay heat transfer during flame exposure. However, because direct thermal-conductivity or thermal-resistance measurements were not performed, this result is discussed as a qualitative thermal-barrier effect rather than a quantitative thermal-insulation property. The backside temperature rise curve (Figure 6c) shows that, under conditions of similar total thickness, both the temperature rise rate and the steady-state temperature of LANM are lower than those of Monolithic ANM, indicating that the interlayer air gap increases the equivalent thermal resistance. The interlayer air gaps reduce continuous solid-phase heat conduction across the thickness, which delays heat penetration through the membrane. Infrared thermal imaging results from hot-water-bath heating corroborate this (Figure 6d): blank substrates exhibited the highest temperature rise, followed by Monolithic ANM, with LANM showing the lowest temperature increase. These findings demonstrate that interlayer air gaps and layered interfaces enhance the material’s equivalent thermal resistance and delay heat penetration, enabling LANM to serve not only for radiative cooling but also for thermal protection.

## 4. Conclusions

In this work, layered alumina nanofiber membranes (LANM) were fabricated via dual-nozzle electrospinning with programmed alternating deposition. This approach introduced interlayer air gaps and periodic interfaces within the membrane without altering the material composition, thereby simultaneously regulating solar-band scattering and bending load transfer. This achieved simultaneous improvement in radiative cooling performance and bending deformability. Under comparable total thickness conditions, solar reflectance increased from 87% (Monolithic ANM) to 95% (LANM), while maintaining a high emissivity of approximately 92% within the atmospheric window. Outdoor testing demonstrated a maximum temperature difference of roughly 5.8 °C for LANM, representing a further reduction of about 2.4 °C compared to Monolithic ANM. Mechanically, weak interlayer interfaces induce a multiple-neutral-axis mechanism and segmented stress transfer, transforming brittle fracture into a more gradual progressive failure mode during bending. It should be noted that this improvement mainly refers to enhanced bendability and higher apparent bending strain, rather than increased nominal bending strength. Maximum bending strain increased significantly from 0.5% to 2.1%.

Additionally, LANM exhibits good morphology retention under direct flame exposure and a thermal-barrier effect under the present testing conditions. The LANM remained intact after 80 s of direct flame torch and showed a lower backside temperature rise. The results indicate that periodic interlayer interfaces and air gaps constructed through spatially partitioned deposition can simultaneously regulate solar-band scattering pathways and bending load transfer processes within a single alumina system. This enables simultaneous regulation of optical response and bending deformation behavior, while maintaining comparable total thickness.

## Data Availability

The original contributions presented in this study are included in the article/Supplementary Material. Further inquiries can be directed to the corresponding author.

## Supplementary Materials

The following supporting information can be downloaded at: https://www.mdpi.com/article/10.3390/ma19132914/s1, Text S1: Derivation of the multilayer bending model; Figure S1: Fiber diameter distribution of the five-layer precursor membrane corresponding to LANM; Figure S2: Fiber diameter statistics for the monolayer precursor Fiber membrane corresponding to Monolithic ANM; Figure S3: Shows the statistical distribution of Fiber diameters after calcination for Monolithic ANM; Figure S4: Schematic diagram of the electromagnetic force-compensated threepoint bending stress–strain testing system; (b) Photograph of the testing apparatus; Figure S5: Repeated outdoor radiative cooling test performed on 7 December 2025; Figure S6: Original load–displacement curves; Figure S7: Three-point bending stress–strain curves of physically stacked paper sheets with different layer numbers (one to four layers); Figure S8: Three-point bending stress–strain curves of physically stacked plaster sheets with different layer numbers (one to four layers); Figure S9: Three-point bending stress–strain curves of physically stacked mica plate laminates with different layer numbers (one to seven layers); Figure S10: Three-point bending stress–strain curves of physically stacked SiO_2_ nanofiber membranes with different layer numbers (one to four layers); Figure S11: Three-point bending stress–strain curves of physically stacked PZT nanofiber membranes with different layer numbers (one to four layers); Figure S12: Fire resistance and thermal insulation performance. (a,b) Comparison of combustion behavior under alcohol lamp flame exposure: (a) PA6 fiber membrane; (b) TPU membrane. (c) Schematic of flame gun test:Direct exposure of aluminum foil versus exposure with a LANM isolation layer; Table S1: Comparison of cooling and mechanical performance between LANM and representative radiative cooling materials; Video S1: Dynamic fracture comparison of Monolithic ANM and LANM under identical bending operation. Refs. [41,42] are included in Supplementary Materials.
