# Supplementary material for: Simultaneous Improvement of Bendability and Passive Daytime Radiative Cooling Performance in Multilayer Alumina Fiber Membranes
Source: Materials (Basel). 2026 Jul 7;19(13):2914. doi: 10.3390/ma19132914 (PMC13362610; doi:10.3390/ma19132914)
Supplement: Supplementary file 1 [file materials-19-02914-s001.zip › Supporting Information.pdf]

Article

Simultaneous Improvement of Bendability and Passive Daytime Radiative Cooling Performance in Multilayer Alumina Fiber Membranes

Yating Zhuang [1,†], Chongyang Fu [1,†], Benxing Guo [1], Weihao Zhai [1], Xueting Ren [1], Depeng Fu [1], Xianchao Li [2], Guangzheng Wang [1], Qizheng Li [1], Yidan Xiao [1], Shuye Zhang [3,*], Hanbin Wang [4,*] and Xiaoxiong Wang [1,*]

1 College of Physics Science, Qingdao University, Qingdao 266071, China
2 Qingdao Xinanqu Refractories Co., Ltd., Qingdao 266000, China
3 State Key Laboratory of Advanced Welding and Joining, Harbin Institute of Technology, Harbin 150001, China
4 Microsystem & Terahertz Research Center, China Academy of Engineering Physics, Chengdu 610200, China
* Correspondence: syzhang@hit.edu.cn (S.Z.); wanghanbin@mtrc.ac.cn (H.W.); wangxiaoxiong@qdu.edu.cn (X.W.)
† These authors contributed equally to this work.

Contents

S1. Derivation of the multilayer bending model
S2. Supplementary Figures
S3. Supporting Video
S4.Table S1. Comparison of cooling and mechanical performance between LANM and representative radiative cooling materials.

Text S1. Derivation of the multilayer bending model

To convert the applied load into bending stress, the elastic stage is approximated using uniaxial loading and small-deflection beam bending, with the elastic modulus E assumed to remain constant. According to Hooke's law, the normal stress at a distance y from the neutral axis is

$$\sigma = \frac{E}{\rho} y \tag{S1}$$

where σ is the normal stress, ρ is the bending curvature radius, and y is the distance from the neutral axis. The maximum stress occurs at $\mid y \mid = y_{\max}$, giving

$$\sigma_{max} = \frac{E}{\rho} y_{max} \tag{S2}$$

According to moment equilibrium, the internal bending moment equals the externally applied bending moment,

$$M = \frac{FL}{2} = \int y\sigma dB \tag{S3}$$

where F is the applied load and L is the span between the two supports. For a rectangular membrane with width b and total thickness H, the maximum bending stress under three-point bending can be written as

$$\sigma_{max} = \frac{3FL}{dh^2} \tag{S4}$$

Thus, the experimental load can be converted into bending stress, which is further used to construct the bending stress–strain curves in the main text.

To understand the mechanical consequence of layer subdivision, consider a membrane with a fixed total thickness H, subdivided into N identical sublayers. The thickness of each sublayer is

$$h = \frac{H}{N} \tag{S5}$$

Under ideal layered conditions, each sublayer bends around its own local neutral plane. According to the bending strain relation,

$$\varepsilon = \frac{y}{\rho} \tag{S6}$$

where y is the distance from the neutral plane and ρ is the bending radius. For a monolithic structure, the outermost distance to the neutral plane is

$$y_{\text{mono}} = \frac{H}{2} \tag{S7}$$

and the corresponding maximum bending strain is

$$\varepsilon_{\text{mono}} = \frac{H}{2\rho} \tag{S8}$$

For an N-layer structure, the outermost distance from the local neutral plane within each sublayer is

$$y_{\text{lami}} = \frac{h}{2} = \frac{H}{2N} \tag{S9}$$

so that the maximum local bending strain in each sublayer becomes

$$\varepsilon_{\text{lami}} = \frac{H}{2N\rho} = \frac{1}{N}\varepsilon_{\text{mono}} \tag{S10}$$

Combining this relation with Hooke's law,

$$\sigma = E\varepsilon \tag{S11}$$

gives

$$\sigma_{\text{lami}} = E\varepsilon_{\text{lami}} = \frac{1}{N}\sigma_{\text{mono}} \tag{S12}$$

This result indicates that, under fixed total thickness and identical bending radius, the maximum local bending stress in a subdivided layered structure decreases to $1/N$ of that of the monolithic structure.

To further estimate the structural failure limit, let the intrinsic fracture stress and intrinsic fracture strain of the material be σ_f and

$$\varepsilon_f = \frac{\sigma_f}{E} \tag{S13}$$

respectively. Failure occurs when the most critical position within a sublayer first satisfies

$$\sigma_{\text{lami}} = \sigma_f \tag{S14}$$

Substituting Equation (S12) into Equation (S14) gives

$$\sigma_{\text{mono}} = N\sigma_f \tag{S15}$$

and the corresponding apparent maximum bending strain of the whole laminate, defined on the basis of the total thickness, can be written as

$$\varepsilon_{\text{max}}(N) = \frac{\sigma_{\text{mono}}}{E} = \frac{N\sigma_f}{E} = N\varepsilon_f \tag{S16}$$

Therefore, although local fracture in each sublayer still occurs at the intrinsic strain ε_f, the apparent maximum bending strain of the entire layered structure increases linearly with the layer number N. This scaling relation provides the theoretical basis for the extrapolated trend shown in Figure 5c of the main text.

S2. Supplementary Figures

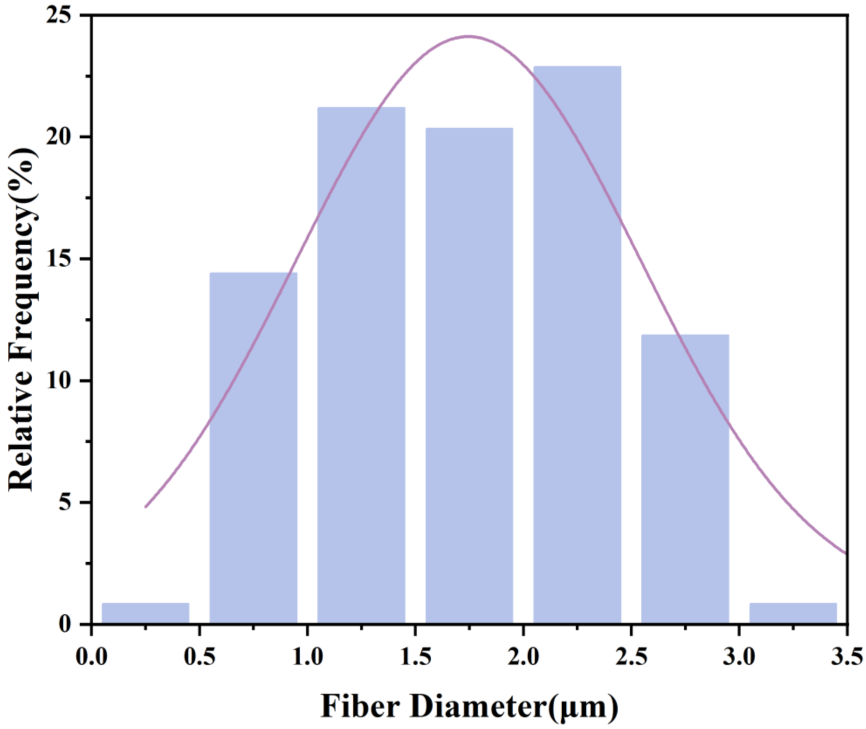

Figure S1. Fiber diameter distribution of the five-layer precursor membrane corresponding to LANM.

The Fiber diameters are predominantly unimodal, with an average diameter of 1.74 ± 0.10 μm, indicating a relatively uniform size distribution of the precursor Fibers.

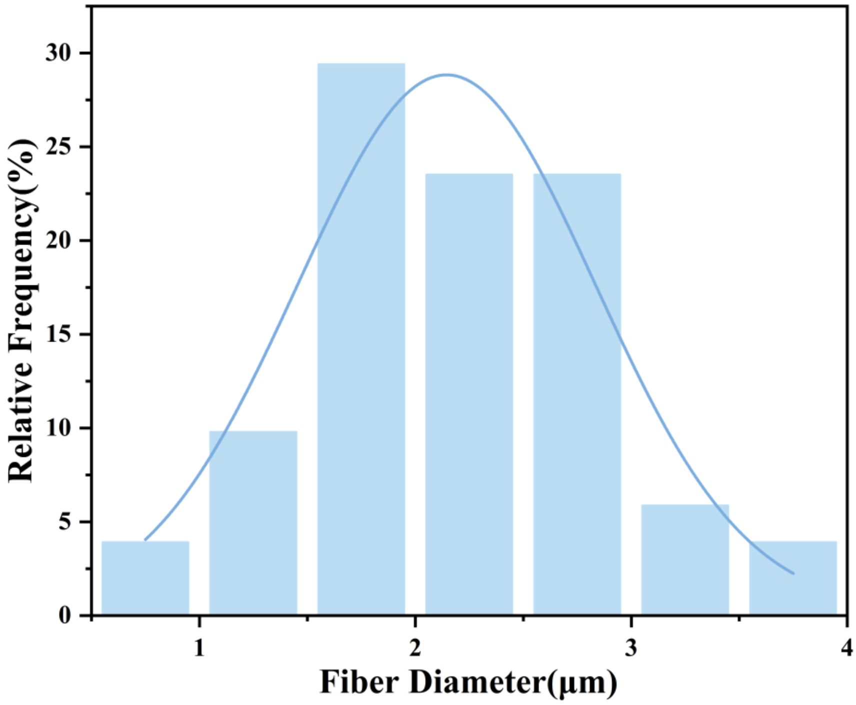

Figure S2 Fiber diameter statistics for the monolayer precursor Fiber membrane corresponding to Monolithic ANM.

The diameter distribution exhibits a unimodal profile with an average diameter of 2.144 ± 1.37 μm, indicating relatively uniform dimensions of the monolayer precursor Fibers.

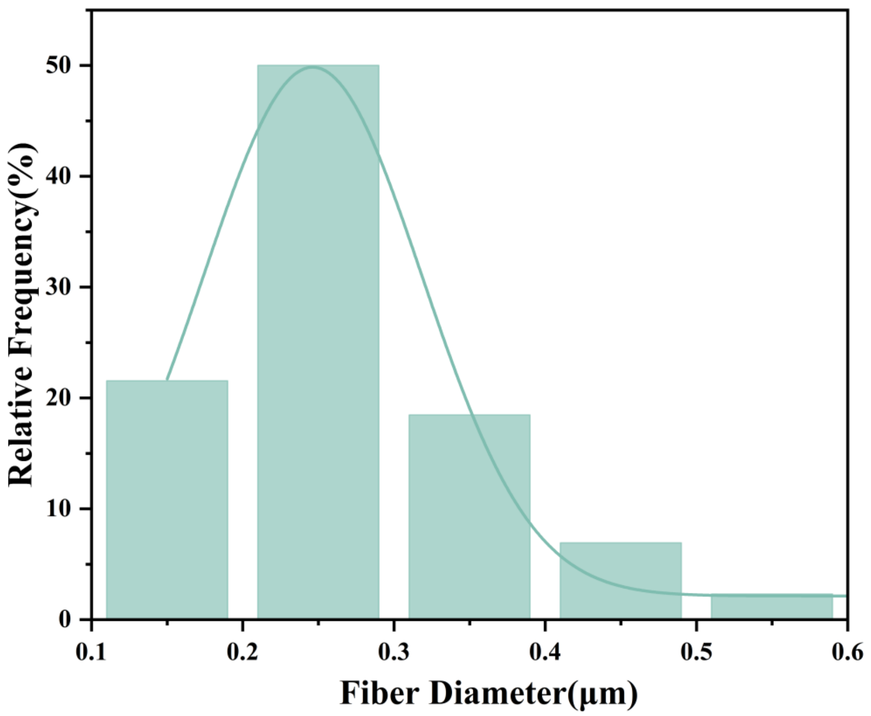

Figure S3 shows the statistical distribution of Fiber diameters after calcination for Monolithic ANM.

The calcined Fiber diameters are concentrated in the submicron range, with an average diameter of 0.23 ± 0.01 μm and a narrow distribution, indicating highly uniform Fiber size after calcination. Compared to the precursor Fibers in Figure S2, the calcined Fibers exhibit a significant reduction in diameter, consistent with the removal of organic components and the densification and shrinkage of the inorganic framework during calcination.

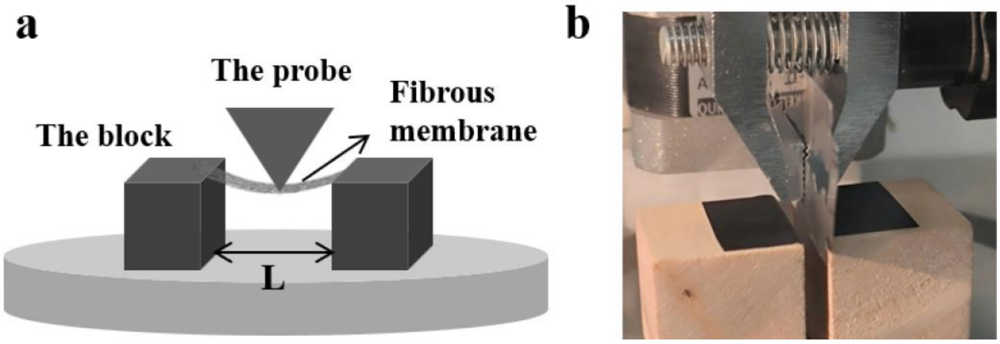

Figure S4. (a) Schematic diagram of the electromagnetic force-compensated threepoint bending stress–strain testing system; (b) Photograph of the testing apparatus.

Figure S4 illustrates the three-point bending testing apparatus and loading method for macroscopically flexible inorganic nanofiber membranes.

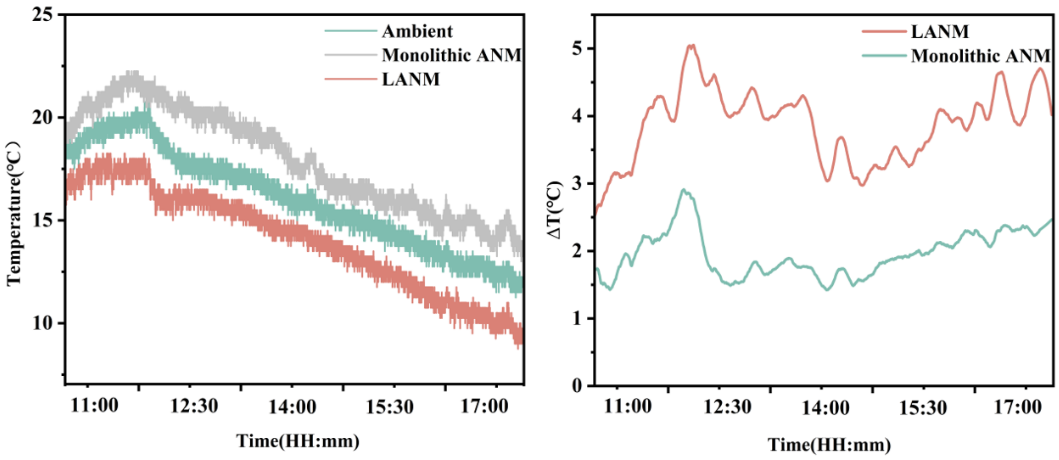

Figure S5. Repeated outdoor radiative cooling test performed on December 7, 2025.

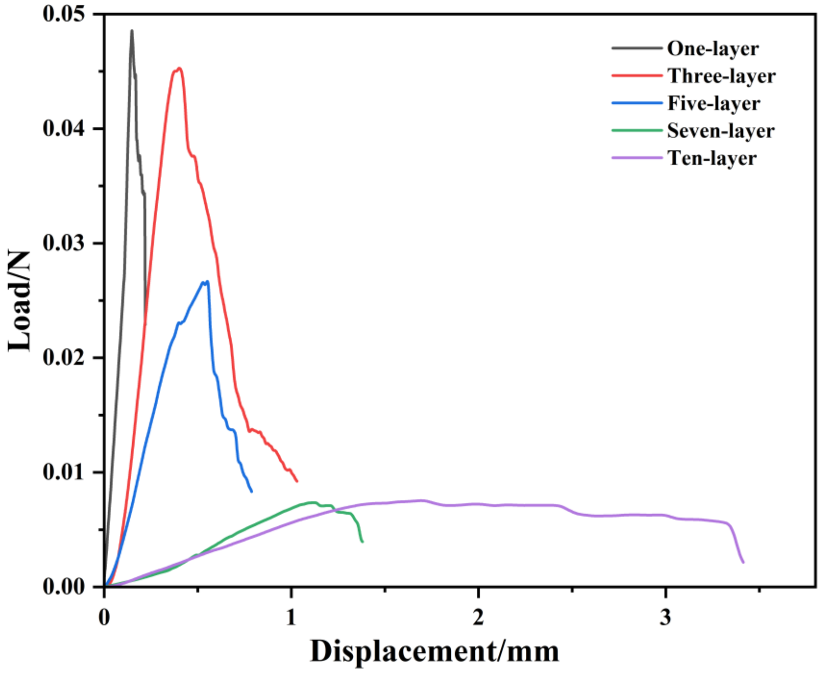

Figure S6. Original load–displacement curves

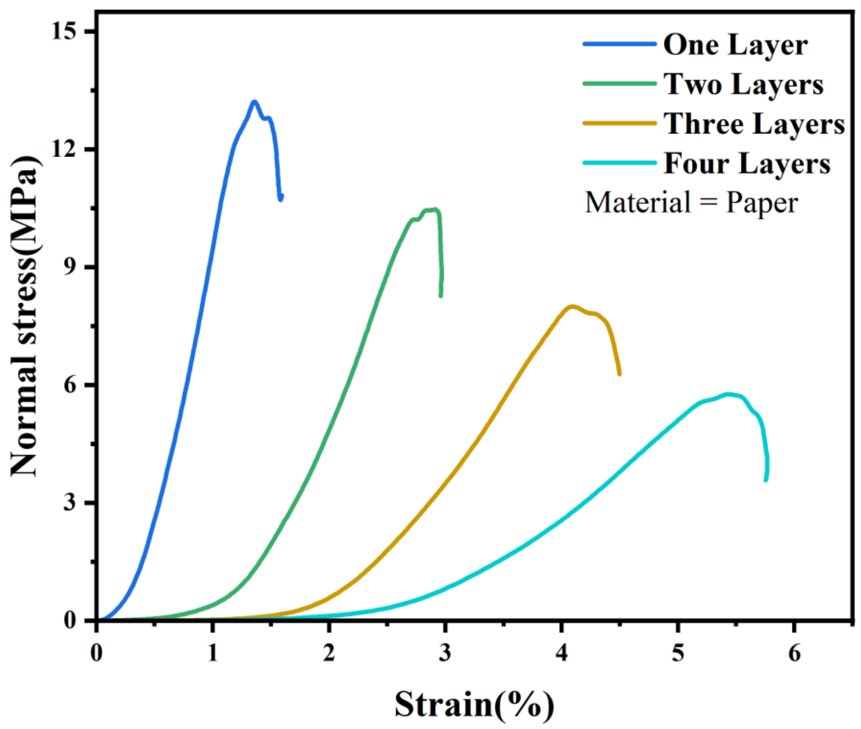

Figure S7. Three-point bending stress–strain curves of physically stacked paper sheets with different layer numbers (one to four layers).

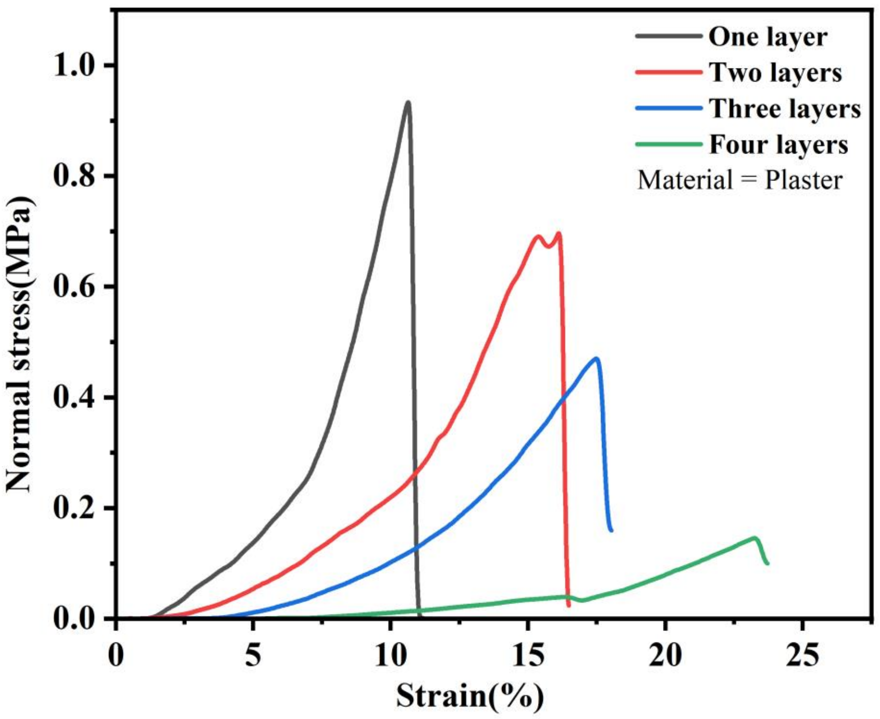

Figure S8. Three-point bending stress–strain curves of physically stacked plaster sheets with different layer numbers (one to four layers).

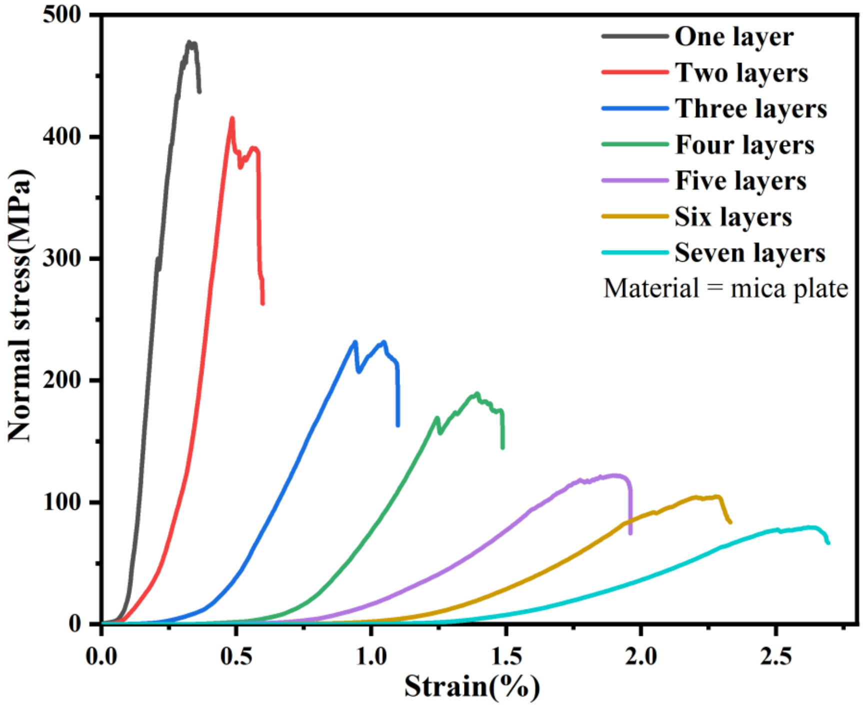

Figure S9. Three-point bending stress–strain curves of physically stacked mica plate laminates with different layer numbers (one to seven layers).

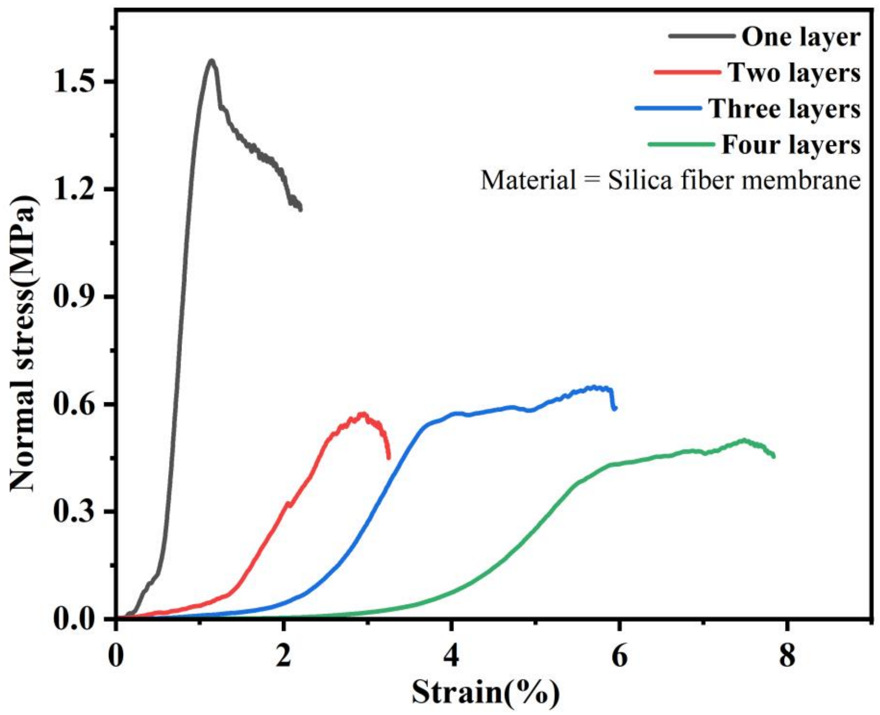

Figure S10. Three-point bending stress–strain curves of physically stacked SiO_2 nanofiber membranes with different layer numbers (one to four layers).

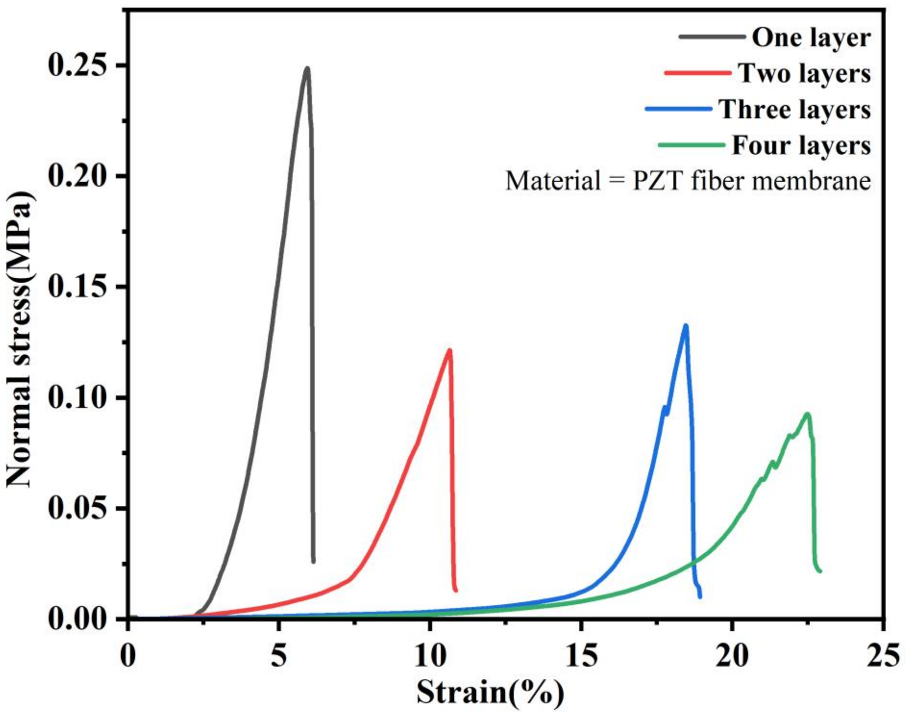

Figure S11. Three-point bending stress–strain curves of physically stacked PZT nanofiber membranes with different layer numbers (one to four layers).

As shown in Figures S5–S9, materials with different compositions exhibit consistent bending response trends when stacked into multilayers: increasing the number of layers reduces the peak bending stress but significantly increases the maximum bending strain. This indicates that the toughening effect arising from “multilayer interfaces” is a universal phenomenon across materials.

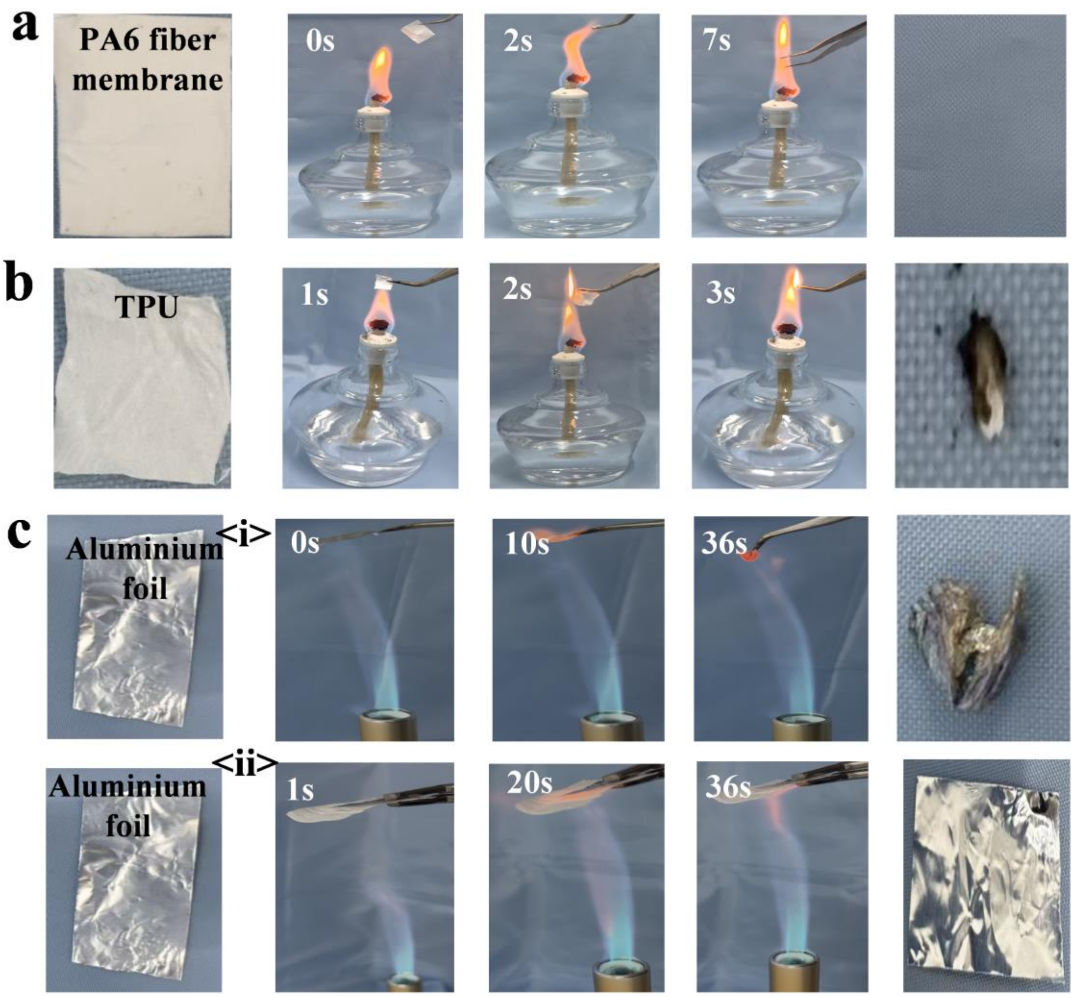

Figure S12. Fire resistance and thermal insulation performance. (a–b) Comparison of combustion behavior under alcohol lamp flame exposure: (a) PA6 fiber membrane; (b) TPU membrane. (c) Schematic of flame gun test:Direct exposure of aluminum foil versus exposure with a LANM isolation layer

S3. Supporting Video

Supporting Video S1. Dynamic fracture comparison of Monolithic ANM and LANM under identical bending operation.

S4. Table S1. Comparison of cooling and mechanical performance between LANM and representative radiative cooling materials.

Material	Cooling performance	Mechanical performance	Ref.
LANM	5.8 °C	2.1 / 0.5 MPa	this work
ZrO_2–Al_2O_3 (sh-ZANF)	6.6 °C	Mechanical strength: 2.48 MPa; bending strain not reported	[1]
SiO_2 NF	7–8 °C	0.6/3.7 MPa	[2]
Al_2O_3 NF	4.5 °C	0.38 / 0.57 MPa*	[3]
porous Si_3N_4–BN ceramic	5.14 °C	flexural strength 31.07 MPa; compressive strength 65.36 MPa	[4]
porous Al_2O_3 ceramic	3.8 °C	0.14/110 MPa	[5]

Note: The mechanical data are listed as reported in the corresponding references. Because different studies used different testing methods, including bending tests, tensile tests, flexural-strength tests, and breaking-strength measurements, these values are provided for qualitative comparison rather than direct one-to-one mechanical benchmarking.

[1] D-C Chen, C-W Hwang, C Y Chang, C-L Kuo, H-L Chen, P-H Lan, M-T Tsai, T-W Wang, D Wan (2025) Superdurable, Flexible Ceramic Nanofibers for Sustainable Passive Radiative Cooling. ACS Nano 19: 28280-28294. https://doi.org/10.1021/acsnano.5c05958

[2] Y Liu, W Zhai, Q Wang, Y Xin, Y Deng, C Fu, S Hui, X Wang, H Wang, S Zhang (2025) Development of an Inorganic Weather-Resistance Composite Fabric for Efficient Passive Daytime Radiative Cooling Applications. ACS Applied Materials & Interfaces. https://doi.org/10.1021/acsami.5c19477

[3] Y Xin, Q Wang, C Fu, S Du, L Hou, X Wei, H Wang, X Wang (2024) Alumina Fiber Membrane Prepared by Electrospinning Technology for Passive Daytime Radiative Cooling. Advanced Functional Materials 35. https://doi.org/10.1002/adfm.202413813

[4] J Zhao, Q Meng, Y Li, Z Yang, J Li (2023) Structural Porous Ceramic for Efficient Daytime Subambient Radiative Cooling. ACS Applied Materials & Interfaces 15:

47286-47293. https://doi.org/10.1021/acsami.3c10772
[5] K X Lin, S R Chen, Y J Zeng, T C Ho, Y H Zhu, X Wang, F Y Liu, B L Huang, C Y H Chao, Z K Wang, C Y Tso (2023) Hierarchically structured passive radiative cooling ceramic with high solar reflectivity. Science 382: 691-697. https://doi.org/10.1126/science.adi4725
